# Prodigiosin-Emerged PI3K/Beclin-1-Independent Pathway Elicits Autophagic Cell Death in Doxorubicin-Sensitive and -Resistant Lung Cancer

**DOI:** 10.3390/jcm7100321

**Published:** 2018-10-03

**Authors:** Wei-Jun Chiu, Shian-Ren Lin, Yu-Hsin Chen, May-Jwan Tsai, Max K. Leong, Ching-Feng Weng

**Affiliations:** 1 Department of Life Science and Institute of Biotechnology, National Dong Hwa University, Hualien 97401, Taiwan; zidane538@gmail.com (W.‐J.C.); d9813003@gms.ndhu.edu.tw (S.‐R.L.); kb5634@yahoo.com.tw (Y.‐H.C.); leong@gms.ndhu.edu.tw (M.K.L.); 2 Neural Regeneration Laboratory, Neurological Institute, Taipei Veterans General Hospital, Taipei 11217, Taiwan; mjtsai2@vghtpe.gov.tw; 3 Department of Chemistry, National Dong Hwa University, Hualien 97401, Taiwan

**Keywords:** prodigiosin, doxorubicin resistant, lung cancer, intratracheal inoculation

## Abstract

Prodigiosin (PG) belongs to a family of prodiginines isolated from gram-negative bacteria. It is a water insoluble red pigment and a potent proapoptotic compound. This study elucidates the anti-tumor activity and underlying mechanism of PG in doxorubicin-sensitive (Dox-S) and doxorubicin-resistant (Dox-R) lung cancer cells. The cytotoxicity and cell death characteristics of PG in two cells were measured by MTT assay, cell cycle analysis, and apoptosis/autophagic marker analysis. Then, the potential mechanism of PG-induced cell death was evaluated through the phosphatidylinositol-4,5-bisphosphate 3-kinase-p85/Protein kinase B /mammalian target of rapamycin (PI3K-p85/Akt/mTOR) and Beclin-1/phosphatidylinositol-4,5-bisphosphate 3-kinase-Class III (Beclin-1/PI3K-Class III) signaling. Finally, in vivo efficacy was examined by intratracheal inoculation and treatment. There was similar cytotoxicity with PG in both Dox-S and Dox-R cells, where the half maximal inhibitory concentrations (IC_50_) were all in 10 μM. Based on a non-significant increase in the sub-G_1_ phase with an increase of microtubule-associated proteins 1A/1B light chain 3B-phosphatidylethanolamine conjugate (LC3-II), the cell death of both cells was categorized to achieve autophagy. Interestingly, an increase in cleaved-poly ADP ribose polymerase (cleaved-PARP) also showed the existence of an apoptosis-sensitive subpopulation. In both Dox-S and Dox-R cells, PI3K-p85/Akt/mTOR signaling pathways were reduced, which inhibited autophagy initiation. However, Beclin-1/PI3K-Class III downregulation implicated non-canonical autophagy pathways were involved in PG-induced autophagy. At completion of the PG regimen, tumors accumulated in the mice trachea and were attenuated by PG treatment, which indicated the efficacy of PG for both Dox-S and Dox-R lung cancer. All the above results concluded that PG is a potential chemotherapeutic agent for lung cancer regimens regardless of doxorubicin resistance.

## 1. Introduction

For many years in the United States, lung cancer and breast cancer have ranked first and second, respectively, for new cancer cases and deaths. For lung cancer in particular, it is estimated that there are fewer new cases than breast cancer, but the estimated number of deaths is almost 4 times higher than breast cancer [1]. According to the origin and morphology of cancer cells, lung cancer can be classified as small cell lung carcinoma (SCLC) or non-small cell lung carcinoma (NSCLC), with 80–85% of lung cancer patients categorized as NSCLC [1,2,3]. A previous study identified cigarette smoking as the major cause of lung cancer [4,5]. However, recent statistical results showed that 52% of lung cancer patients are non-smokers, especially in women [6]. Moreover, non-significant symptoms and alteration of lung cancer epidemiology increases the difficulty of lung cancer diagnosis [7] and consequently influences the timing of treatment and survival rates.

In clinics, surgery, chemotherapy, radiation therapy, and adjuvant therapy have been executed the strategies for treating cancer patients, all of which, ideally, result in killing cancer cells [2]. Liposomal-doxorubicin and docetaxel combined with radiotherapy has been used for NSCLC treatment as well [8]. However, chemotherapeutic agents are mostly non-tissue specific, which affects the lungs and other organs such as the liver, kidney, bone marrow, and heart [9]. Therefore, new formulations of chemotherapeutic agents, for example, nanoparticle encapsulation, adjuvant therapy for enhancing chemotherapeutic efficacy, and new natural tumoricidal agents such as Ocoxin, Panaxynol, and sulforaphane have been developed to overcome the limitations of chemotherapeutic agents and the high mortality of lung cancer [10,11,12,13,14].

Prodigiosin (PG, PubChem ID 5351169) is a red fluorescent pigment that was discovered in the 1970s, and it is the first member of tripyrrole compound prodiginines [15]. Numerous studies have demonstrated anti-cancer activity in various types of cancer [16]. In breast cancer, PG could activate apoptosis by different signaling pathways including reducing Wnt/β-catenin, c-Jun N-terminal kinases/p38/RAD51 (JNK/p38/RAD51), or glycogen synthase kinase 3 beta/NAG-1 (GSK-3β/NAG-1), and inducing endoplasmic reticulum (ER) stress [17,18,19,20]. As well as inducing apoptosis, PG could also enhance paclitaxel cytotoxicity via down-regulating surviving expression and cause cell death in multidrug-resistant cell lines via mitochondria-mediated apoptosis [21,22,23]. In the apoptotic mechanisms of PG against colorectal cancer, c-Jun N-terminal kinase/truncated p73 isoform (c-Jun/ΔNp73) and lysosome acidification were inhibited [24,25,26,27,28]. It has been reported that the apoptosis of PG-treated hepatocellular carcinoma cells was caused by targeting surviving [29]. Also, it has been shown that PG was able to induce autophagy in oral squamous cell carcinoma cells by down-regulating the protein kinase B/mammalian target of the rapamycin (Akt/mTOR) signaling pathway [30]. However, the beneficial effects and potential mechanisms of PG on NSCLC remain to be explored. This study appraises the potential of PG as a regimen for lung cancer, especially for doxorubicin-sensitive (Dox-S) and doxorubicin-resistant (Dox-R) lung cancer. Also, the mechanical action that PG triggers in these two lung cancer cells is elucidated.

## 2. Experimental Section

### 2.1. Reagents and Chemicals

PG was provided by Prof. Jui-Hsin Su (Institute of Marine Biotechnology, National Dong Hwa University, Pingtung, Taiwan). All cell-culture reagents were obtained from Thermo-Fisher (Waltham, MA, USA) and other chemicals from Sigma-Aldrich (St. Louis, MO, USA).

### 2.2. Cell Culture and Induction of Doxorubicin Resistance

A549 (Dox-S) cells were obtained from Bioresource Collection and Research Center (BCRC, Hsinchu, Taiwan) and cultured in Dulbecco’s minimum essential medium (DMEM) containing 10% FBS and 1% penicillin/streptomycin, and the culture medium was renewed every 2 days. Cells were placed under 37°C, 5% CO_2_ and sub-cultured by 0.25% trypsin/ethylenediaminetetraacetic acid (EDTA) until80–90% confluence. For uniformity and consistency, all cell-based experiments were carried out within 20 passages.

Anti-Dox-A549 (Dox-R) cells were derived from parental A549 cells by pulsing treatment of doxorubicin (Dox). In brief, A549 cells were incubated with 2 μM of Dox for 24 h followed by regular culture conditions for 1 week. The previous step was repeated with higher Dox concentrations until 100 μM was reached.

### 2.3. Cytotoxicity Assay

The cytotoxicity of PG and Dox were tested via MTT assay. Cells were inoculated every 7 × 10^3^ cells in 96-well plates and incubated overnight under culture conditions. Cells were treated with 1–25 μM of Dox or 2.5–100 μM of PG for 24 h, respectively. After incubation, 20 μL of 25 μg/mL MTT solution was added and further incubated for 4 h. The purple crystal was solved by dimethyl sulfoxide and the optical intensity of 570 nm in each well was measured by Opsys MR Microplate Reader (Thermo-Fisher, MA, USA). Cytotoxicity was represented by cell viability, which is the percentage difference between treated and untreated groups. 

### 2.4. Cell Cycle Analysis

Cell cycle analysis detects DNA content within cells by staining them with fluorescent dye propidium iodide (PI). 7 × 10^4^ cells/well of cells were seeded into 12-well plates and incubated overnight under culture conditions. Cells were incubated with various concentrations of PG and Dox for 24 h, respectively. Then, cells with a culture medium were harvested by trypsin/EDTA and washed with warm phosphate-buffered saline (PBS) through centrifuging at 1000 rpm for 10 min. Cells were fixed with 70% ethanol/PBS at −20 °C for at least 3 h and stained by staining solution (20 μg/mL PI, 0.1% Triton X-100, and 0.2 mg/mL RNase) for 30 min under 37 °C. Finally, DNA content within 10^4^ cells/sample was detected by the Cytomics ^TM^ FC500 flow cytometer (Beckman-Coulter, Brea, CA, USA).

### 2.5. Western Blotting

3.5 × 10^5^ cells/well were seeded into the 6-well plates and incubated until 80% confluence. Cells were treated with PG for 24 h and lyzed using radioimmunoprecipitation (RIPA) buffer after incubation. Proteins were separated using sodium dodecyl sulfate polyacrylamide gel electrophoresis (SDS-PAGE) and subsequently transferred to a polyvinylidene difluoride (PVDF) membrane (Millipore, Bedford, MA, USA). The desired proteins were stained for appropriate 1st and 2nd antibodies and detected by LAS-3000 (FUJIFILM, Tokyo, Japan) after soaking with enhanced chemiluminescence (ECL) reagents. Chemiluminescent intensity of each protein was normalized with glyceraldehyde 3-phosphate dehydrogenase (GAPDH) and represented as a ratio to the control.

### 2.6. Lung Cancer Animal Model Establishment

Animal experiments were approved by the National Dong Hwa University Animal Ethics Committee and the experimental protocols used were according to the “Guide for the Care and Use of Laboratory Animals” of National Dong-Hwa University. We used six males per group, 6–8 week-old mice of C57BL/6; they were weighed, and lung cancer was induced by intra-tracheal (IT) injection. On day 0, 1 × 10^7^ cells/animals of Dox-R and Dox-S were intra-tracheally injected into the mice using pentobarbital anesthetic every 7 days until day 21. Next, 162 μg/kg and 646 μg/kg of PG were IT injected once every 5 days from day 21 to 53. The mice were sacrificed, and the lungs, liver, kidney, and spleen were taken followed by fixing and hematoxylin and eosin staining.

### 2.7. Statistical Analysis

Data were represented as mean ± standard deviation (SD) in at least three independent experiments. The results were analyzed by one-way analysis of variance (ANOVA) followed by Tukey’s test. The differences between tested and control groups (*p* < 0.05) are marked “*” in the histograms. All statistical protocols were performed on GraphPad Prism Ver 7.04 (GraphPad Software, La Jolla, CA, USA).

## 3. Results

### 3.1. Cytotoxicity of PG inDox-S and Dox-R Cells

**Figure 1:**
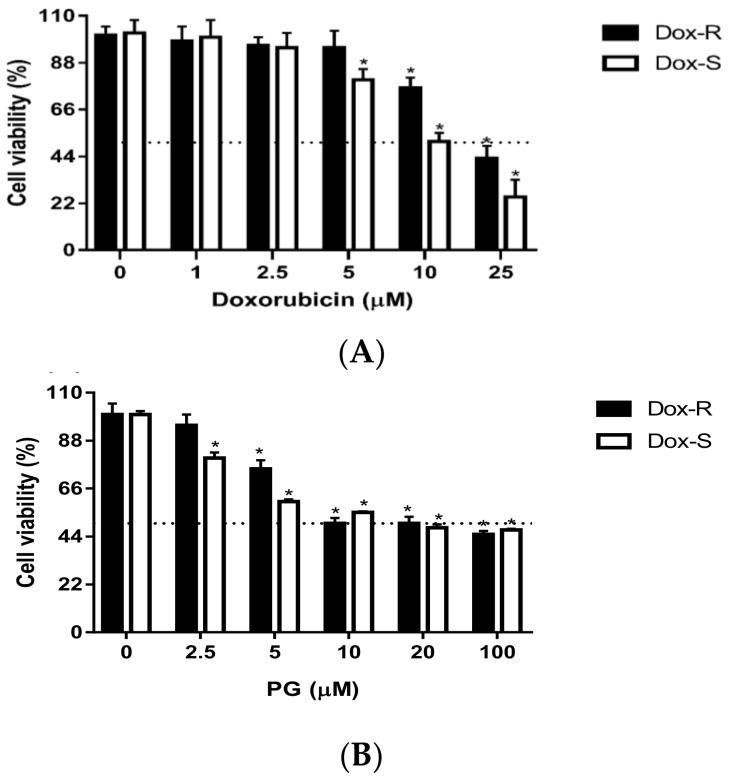
Cytotoxicity of (A) doxorubicin and (B) prodigiosin in A549 and Anti-Dox-A549 cells. A549 (Dox-S) and anti-Dox-A549 cells (Dox-R) were treated with doxorubicin (Dox) and prodigiosin (PG) for 24 h, respectively. Results were represented as mean ± standard deviation (SD) from 3 independent experiments. *, *p* < 0.05 compared with untreated control.

Dox-R and Dox-S cells were exposed to various concentrations of PG and Dox (chemotherapy drug) for measuring the cell viability by MTT assay. Dox-R and Dox-S cells were treated with 2.5–100 μM of PG and 1.0–25 μM of Dox for 24 h. The cytotoxicity of Dox was different in two cells and the half maximal inhibitory concentration (IC_50_) of both Dox-R and Dox-S cells was 25 μM and 10 μM, respectively (Figure 1A). Dox-R was successfully generated and the resistant factor was around 2.5. Interestingly, the cytotoxicity of PG in both Dox-R and Dox-S cells was similar; the IC_50_ of PG were all in 10 μM (Figure 1B). The next experiment was to evaluate the cell death characteristics of PG-induced cytotoxicity.

### 3.2. Cell Death Characteristics in PG-Induced Cytotoxicity

**Figure 2:**
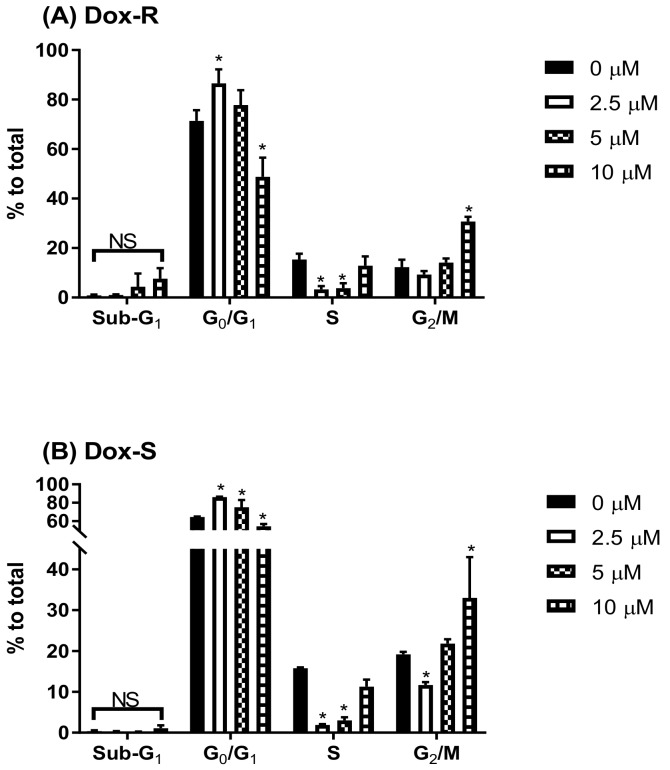
Cell cycle change in Dox-R and Dox-S cells after PG treatment. (**A**) Dox-R and (**B**) Dox-S cells were treated with PG for 24 h, respectively, and stained with propidium iodide for DNA content measurement. Results were shown as mean ± SD from 3 independent experiments. * and NS indicated “*p* < 0.05” and “not significant” when compared with untreated control, respectively.

**Figure 3:**
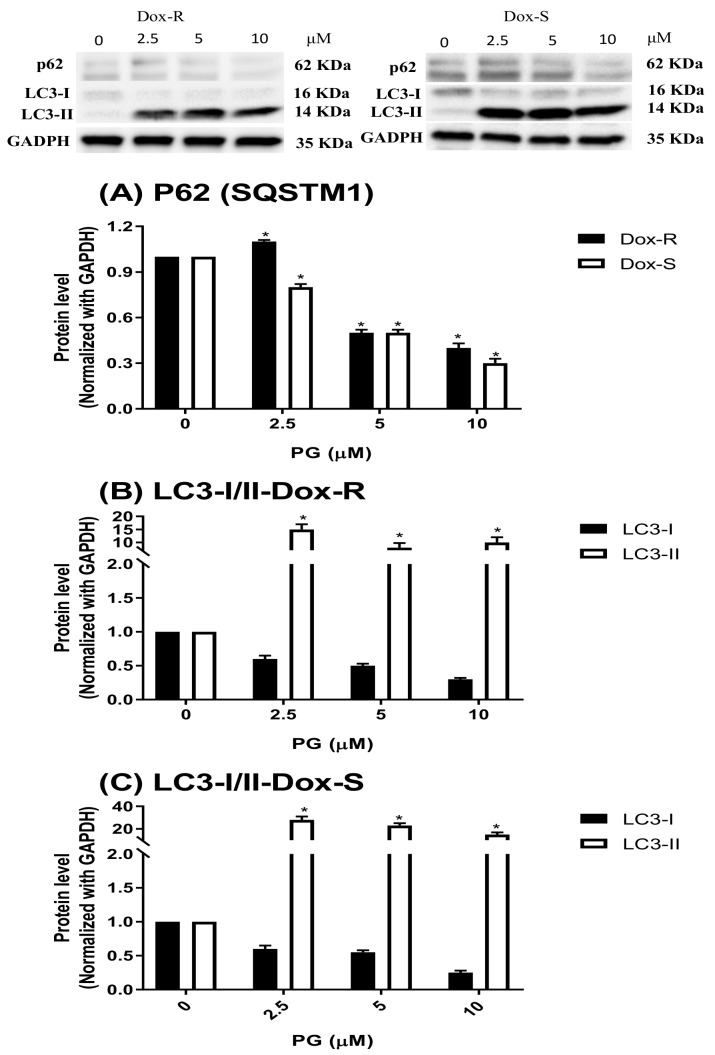
Alteration of autophagic marker levels in Dox-R and Dox-S cells after PG treatment. Protein levels of (**A**) p62 (SQSTM1), LC3-I/II in (**B**) Dox-R and (**C**) Dox-S were analyzed after PG treatment by Western blotting. Protein levels were represented as mean ± SD from 3 independent glyceraldehyde 3-phosphate dehydrogenase (GAPDH)-normalized chemiluminescent experiments. *, *p* < 0.05 compared with untreated control.

**Figure 4:**
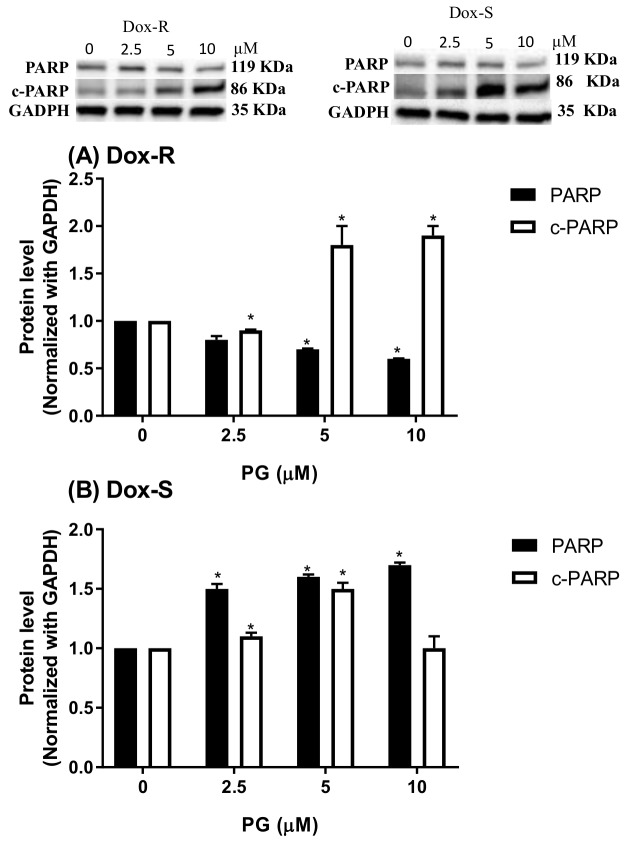
Expression of poly ADP ribose polymerase (PARP) levels in (A) Dox-R and (B) Dox-S cells after PG treatment. Non-cleaved PARP (PARP) and cleaved-PARP (c-PARP) levels after PG treating were measured by Western blotting and all chemiluminescent intensity were normalized with GAPDH. Results were shown as mean ± SD with 3 independent experiment. *, *p* < 0.05 compared with untreated control.

To determine the phase of the cell cycle at PG, Dox-R and Dox-S cells were treated with PG, stained with PI, and fluorescent intensity was analyzed by flow cytometry. Dox-R and Dox-S cells, treated with 10 μM of PG, were found to have a decreased S and G_2_/M phase at low concentrations and showed increases in a dose-dependent manner. When compared with the untreated control, the ratio of Sub-G_1_ phase was insignificantly increased (Figure 2). Also, the LC3-II levels after PG treatment were increased over 10 times, which directly proved the activation of autophagy within 2 cells (Figure 3B,C). In addition, p62 expression levels were down-regulated, which was matched with the elevation of LC3-II (Figure 3A), suggesting that PG can activate autophagy. Additionally, cleaved-poly ADP ribose polymerase (c-PARP) was potentiated, which is a marker of apoptosis activation (Figure 4), from which the differences between poly ADP ribose polymerase (PARP) and c-PARP expression levels in both Dox-R and Dox-S cell lines can be observed, suggesting that PG can induce both apoptosis and autophagy in both cell lines. The autophagy triggered by PG was also confirmed via autophagic inhibitor-bafilomycin A1 (BA1) and 3-methyladenine (3-MA) (data not shown). The subsequent work examined the upstream mechanism of PG-induced autophagy.

### 3.3. Underlying Mechanism of PG-Induced Autophagy

**Figure 5:**
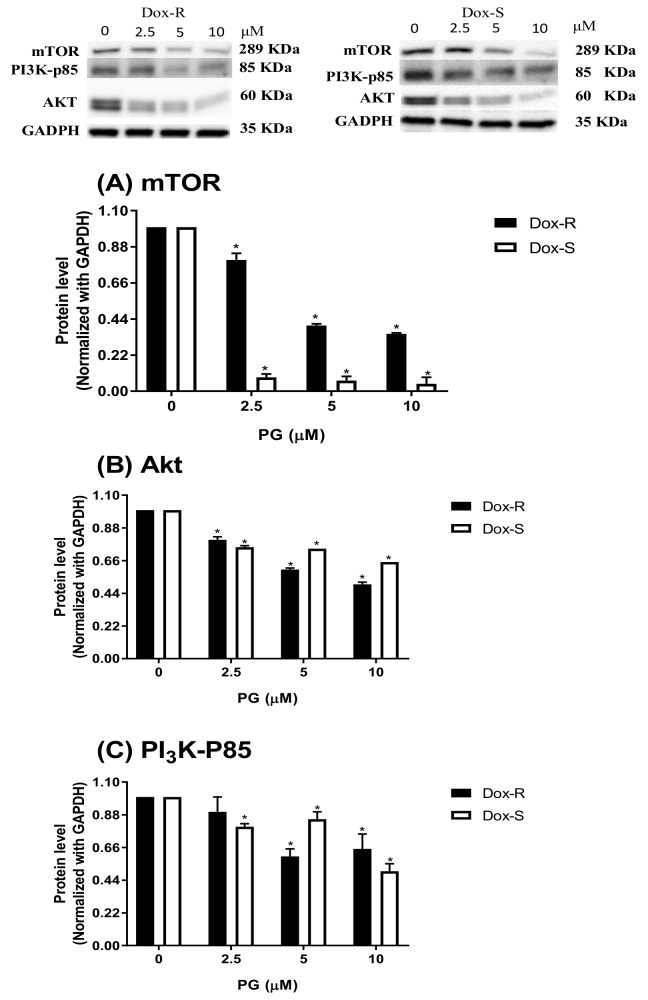
Autophagic inhibitor level change in Dox-R and Dox-S cells after PG treatment. (**A**) mammalian target of rapamycin (mTOR), (**B**) protein kinase B (Akt), and (**C**) phosphatidylinositol-4,5-bisphosphate 3-kinase-p85 (PI3K-p85) protein levels were determined by chemiluminescent Western blotting and normalized with GAPDH. Results were shown as mean ± SD in 3 independent experiments. *, *p* < 0.05 compared with untreated control.

**Figure 6:**
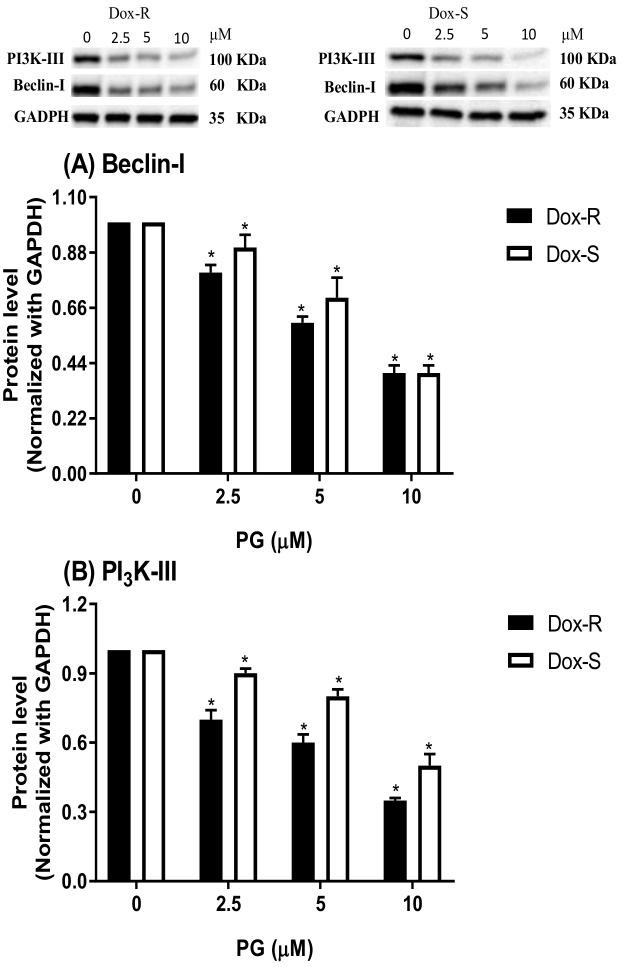
Alteration of autophagic inducer levels in Dox-R and Dox-S cells after PG treatment. (**A**) Beclin-1 and (**B**) PI3K-III were measured by Western blotting and normalized with GAPDH. Results were represented as mean ± SD from 3 independent experiments. *, *p* < 0.05 compared with untreated control.

The regulators of autophagy can be grouped as inducers (activators) or inhibitors (reducers), which counter balance and lead cells to a normal state or autophagy [31]. Upstream regulators include mammalian target of rapamycin (mTOR), phosphatidylinositol-4,5-bisphosphate 3-kinase-p85 (PI3K-p85), and protein kinase B (Akt) as inhibitors, and Beclin-1 and PI3K-Class III (PI3K-III) as inducers [32]. The following section examines the upstream regulating mechanism of PG-induced autophagy. By analyzing mTOR, Akt, and PI3K-p85 levels in PG-treated cells, it was found that these three autophagy inhibitors all reduced expression in a dose-dependent fashion (Figure 5). Surprisingly, two autophagic inducers, Beclin-1 and PI3K-III, were all decreased dose-dependently, which might infer PI3K-Class III/Beclin-1-independent activation to autophagy by PG (Figure 6). The above-mentioned measurement of upstream autophagic regulators showed the possible mechanism of autophagy activation by PG. PG reduced autophagic inhibitor expression and activated non-PI3K-Class III/Beclin-1 inducer expression, which results in the start of autophagy. The last part of the study evaluated the potential efficacy of PG in Dox-R- and Dox-S-bearing in the animal model.

### 3.4. Curing Efficacy of PG in Tumor-Bearing Mice

**Figure 7:**
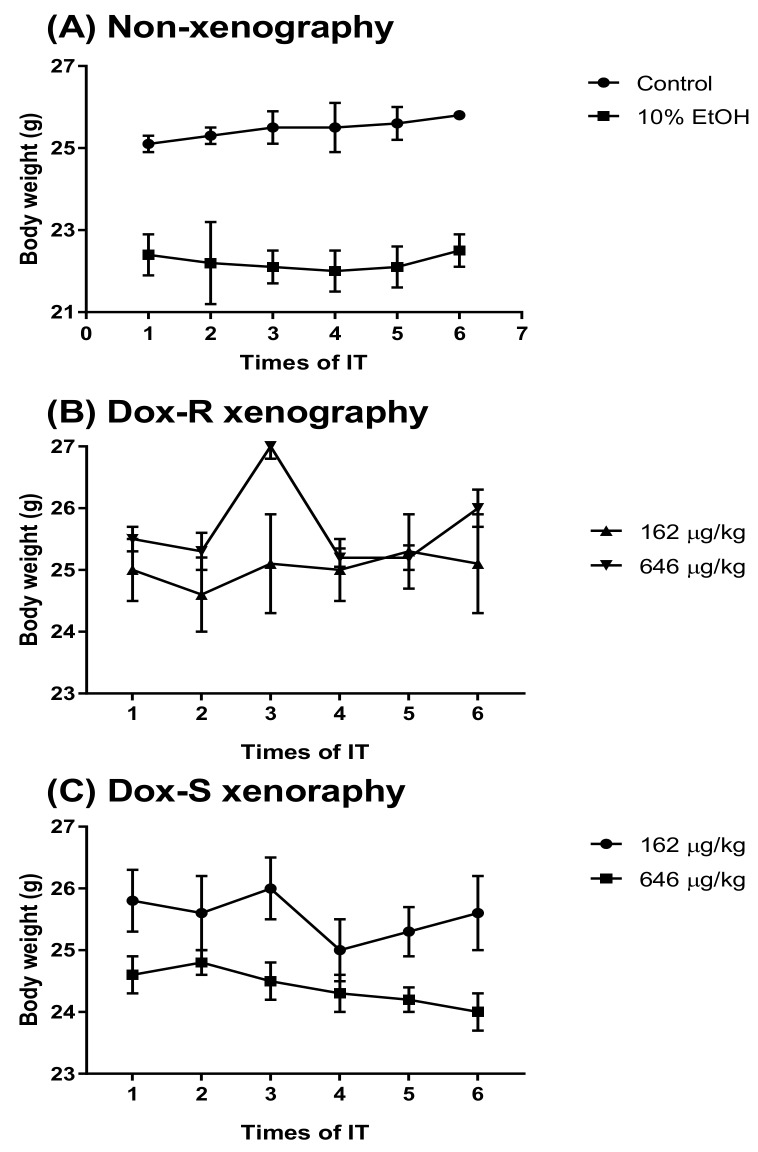
The body weight of the Dox-R and Dox-S xenograft mice during treatment with PG. (**A**) Non-xenograft, (**B**) Dox-R xenograft, and (**C**) Dox-S xenograft mice treatment with PG (162 and 646 μg/kg B. wt) or 10% EOH by intra-tracheal (IT) injection once every 5 days and measured body weight at each time of IT injection. Results were represented as mean ± SD of 6 mice in each group.

**Figure 8:**
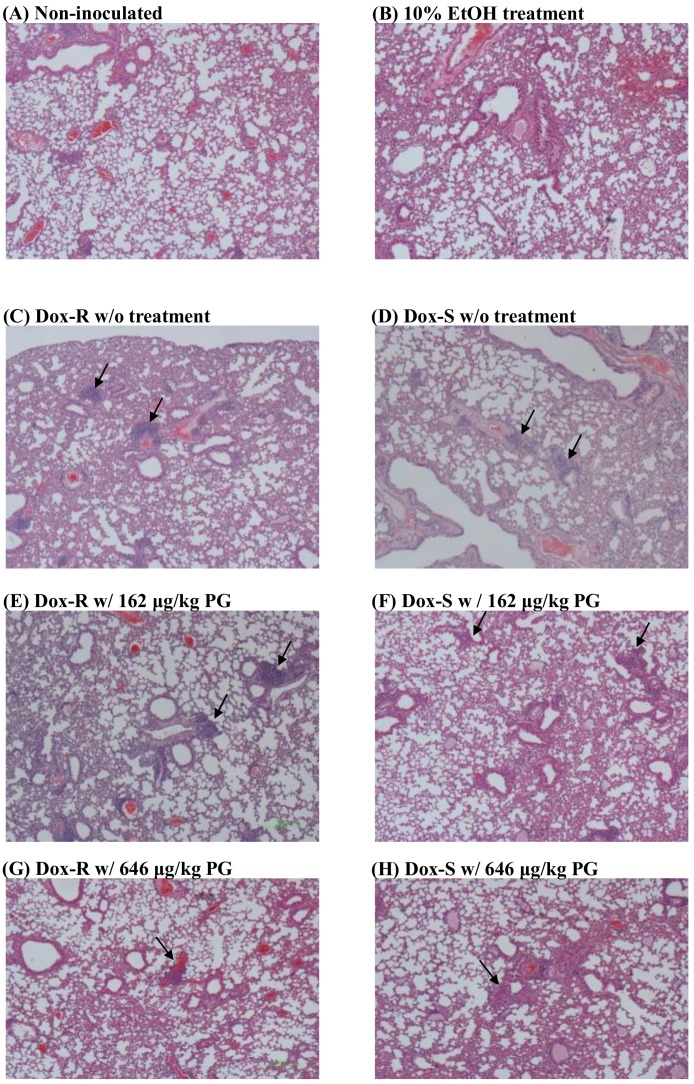
In situ xenograft tumor after PG treatments. Lung tumor (**A**) without tumor injection, with tumor injection and treated with (**B**) 10% EtOH, (**C**,**D**) untreated, (**E**,**F**) 162 μg/kg PG, and (**G**,**H**) 646 μg/kg PG and stained with hematoxylin and eosin. All tissue snapshots were taken in 10 × 10. Black arrow points to the location of tumor cells.

All the above descriptions for cytotoxicity and possible mechanisms of PG against Dox-R and Dox-S cells were demonstrated. The present section describes the curing potential of PG in Dox-R and Dox-S bearing mice. C57Bl/6 mice were intra-tracheally (IT) inoculated with Dox-R and Dox-S cells, and treated with 162 μg/kg and 646 μg/kg of PG, respectively, also via IT once every 5 days. Compared with non-inoculated mice, the body weight of Dox-R and Dox-S inoculated mice was not significantly different during the PG treatment. This result indicated that there was no acute toxicity in treating the mice (Figure 7). After the regimen was completed, the mice were sacrificed and the lungs were removed for sectioning. When compared with normal controls, unstructured cell accumulation around the trachea was identified in Dox-R and Dox-S inoculated mice without treatment, which revealed the successful inoculation of Dox-R and Dox-S cells (Figure 8 A,C,D, black arrow). Following completion of the PG-treating regimen, tumors gathered around the trachea were dose-dependently diminished, and no observable differences between Dox-R and Dox-S cells were apparent (Figure 8E–H). These results showed the treatment efficacy of PG in both Dox-sensitive and Dox-resistant lung cancer; however, this is subject to further validation by more research with more animals. 

## 4. Discussion

This study successfully demonstrated the potential of PG for treating lung cancer by in vitro and in vivo strategies. The cytotoxicity of PG was similar with doxorubicin against Dox-R and Dox-S cells. An insignificant increase in sub-G_1_, LC-3-II, and c-PARP increases elucidated that the cause of PG-induced cell death was both apoptosis and autophagy where autophagy might be the major cause. A study of the fundamental mechanisms showed that PG may activate autophagy via reducing Akt/PI3K/mTOR expression, further through Beclin-1/PI3K-Class III independent pathway, and finally through potentiated LC-3-II cleavage and p62 degradation. In treating Dox-R and Dox-S bearing mice, PG significantly diminished tumor cell accumulation around the trachea of the mice, which proved the efficacy of PG.

Due to poor diagnosis and chemotherapeutic responses, several published studies have attempted to discover new chemotherapeutic candidates from natural sources against lung cancer, particularly NSCLC [33,34,35]. Yellow and green pigments isolated from the seed oil of *Calophyllum inophyllum* L. was found to have tumoricidal activity against colon and lung cancers and potentiated gefitinib cytotoxicity [36]. Another study showed crude extract of *Neosartorya laciniosa* KUFC 7896 and *Neosartorya tsunodae* KUFC 9213 potentiated doxorubicin tumoricidal activity by increasing Dox accumulation in lung cancer nuclei [37]. Of note, natural compounds such as curcumin and β-asarone from the Chinese herb *Acorus tatarinowii* [38,39] have showed their tumoricidal activity by reducing the Wnt/β-catenin signaling pathway, by enhancing current chemotherapeutic drugs, for example, curcumin and phloretin [40,41], and by inducing intrinsic or extrinsic apoptotic pathways, including waltonitone, matrine, oxymatrine, astragalin (kaempferol-3-*O*-β-d-glucoside), and 3-*O*-α-l-arabinosyl oleanolic acid from *Schumacheria castaneifolia* Vahl [42,43,44,45,46]. Some natural compounds’ inhibited growth factor have overcome drug resistance, for example, granulatimide, danorubicin, penicinoline, austocystin D, equol, kaempferol, resveratrol, ellagic acid, and butein [47,48,49]. Isothiocyanates, including benzyl isothiocyanate and phenethyl isothiocyanate, reduced NSCLC metastasis by inducing oxidative stress, but also activated protective autophagy [50,51]. Digitoxigenin monodigitoxoside, a steroid glycoside from *Digitalis lanata* Ehrh., exerted long-term cytotoxicity via binding to Na/K-ATPase [52]. Gigantol from *Dendrobium draconis* Rchb. f. exhibited its antitumor activity by hindering lung cancer metastasis [53]. Moreover, ginkgetin activated autophagic cell death by targeting SQSTM1 [54]. All the above studies point to the potential of natural compounds against lung cancer.

Since PG was identified in the 1970s, it has exhibited anticancer activities in various cancers [16]. However, reports of PG against lung cancer is rare. In the SCLC cell line, GLC4, PG stimulates apoptosis through intrinsic apoptotic pathways, the mitochondrial membrane potential is lost and then cytochrome c is released [55]. Furthermore, in the Dox-R GLC4 cell line, PG also exerts apoptosis via a similar signaling pathway [56]. This current study was the first report of the curing efficacy of PG in both Dox-S and -R NSCLC cells.

In studying the underlying mechanism of PG-induced autophagy, the Beclin-1/PI3K-Class III independent pathway was elicited in this study. According to the previous theory of autophagosome formation, PI3k-Class III is the essential enzyme for producing phosphatidylinositol-3,4-bisphosphate (PtsInd(3,4)P) to form autophagosome membrane and trigger autophagy initiation [57]. Accordingly, Beclin-1 has been described as the key regulator for PI3K-Class III activity [58]. However, other studies have reported that autophagosome could be formed by a group of proteins named autophagy-related proteins (Atg). The Atg-related autophagy cascade is initiated from Atg12 and ends with Atg12/Atg5/Atg16L1 complex, which activates autophagosome nucleation and elongation coupled with LC3 [59]. Recently, numerous studies have been published for elucidation of the Beclin-1 independent autophagy property of natural compounds. *Rhus coriaria* L. extract, which is commonly employed in southern Europe, promotes unfolded protein response and triggered Beclin-1 independent autophagy in colorectal cancer [60]. The major constitutes of ginseng, ginsenoside Rg1, could assist mice against sepsis-associated encephalopathy by inducing Beclin-1 independent autophagy, resulting in promoting the survival rate [61]. Resveratrol has also proved to induce Beclin-1 independent autophagy in HeLa cells through intracellular calcium flux and inositol 1,4,5-triphosphate receptors [62]. Using lung cancer cells as a platform, luteolin (common flavonoid), neferine (from lotus), and 1′S-1′-acetoxychavicol acetate (from *Alpinia conchigera* Griff.) exhibited autophagy-activating properties via a Beclin-1 independent manner [63,64,65]. Our previous study revealed that 16-hydroxy-cleroda-3,13-dien-16,15-olide (HCD) and PG activated Beclin-1 independent autophagy and led to oral squamous cell carcinoma cell death [30,66]. This study confirmed that Beclin-1 independent autophagy by PG was not tissue specific.

A further conclusion of this study is that there is polymorphism in commercial cancer cell lines. Genomic instability in in vivo cancer tissue has been well identified, and is also the origin of cancer cell polymorphism [67]. Polymorphism of cancer cells produces widely diverse phenotypes that can adapt to various stresses such as chemotherapeutic drugs, hypoxia, and nutrient deprivation [68]. In our study, both apoptosis and autophagy were activated in the same cell populations, which clearly indicates that A549 cells were not uniform in phenotype. This result demonstrated the genomic instability of cancer cells, even in commercial cell lines.

## 5. Conclusions

PG can induce apoptotic and autophagic cell death in both Dox-R and Dox-S cells via reducing the Akt/PI_3_K-p85/mTOR signaling pathway. Also, PG-induced autophagy was in a Beclin-1/PI3K-Class III independent manner. This was demonstrated by in vitro cell tests and the tumoricidal activity of PG that was observed in the in vivo mice test. Overall, our study identified the autophagic effect of PG in Dox-R and Dox-S cells, which showed their promising value as chemotherapeutic candidates against lung cancer.
